# Assessment of red blood cell distribution width as a prognostic marker in chronic lymphocytic leukemia

**DOI:** 10.18632/oncotarget.9055

**Published:** 2016-04-27

**Authors:** Monika Podhorecka, Dorota Halicka, Agnieszka Szymczyk, Arkadiusz Macheta, Sylwia Chocholska, Marek Hus, Zbigniew Darzynkiewicz

**Affiliations:** ^1^ Department of Hematooncology and Bone Marrow Transplantation, Medical University of Lublin, Lublin, Poland; ^2^ Department of Pathology, New York Medical College, Valhalla, New York, USA

**Keywords:** CLL, RDW, mTOR, ZAP-70, CD38

## Abstract

Red blood cell distribution width (RDW) is a quantitative measure of the variability in size of circulating erythrocytes. It was recently reported that RDW is a prognostic factor for infection diseases, cardiovascular and pulmonary diseases, as well as some neoplasms. Moreover, RDW is remarkably strong predictor of longevity, including all causes of death, for adults aged 45 years and older. To explain this occurrence it was proposed that persistent IGFs/mTOR signaling is one of the factors that play a role in affecting the RDW and mortality.

The above observations induced us to analyze the prognostic role of RDW in chronic lymphocytic leukemia (CLL) being the most frequent type of adult leukemia in Western countries. The obtained results have shown that RDW may be considered as a potential CLL prognostic marker. Elevated RDW level at the moment of diagnosis was associated with advanced disease and presence of other poor prognostic factors. It is also connected with overall survival indicating shorter time in patients with elevated RDW. It is possible that the presently observed correlation between mortality and RDW of the CLL patients is affected by their metabolic (IGF-1/mTOR driven)- rather than chronological- aging. The patients with high level of RDW are expected to have an increased persistent level of IGF-1/mTOR signaling. Within the framework of personalized therapy, these CLL patients therefore would be expected to be more sensitive to the treatment with mTOR inhibitors.

## INTRODUCTION

Chronic lymphocytic leukemia (CLL) is the most frequently diagnosed leukemia of older patients in Western countries. The disease is characterized by the accumulation of leukemic CD19+/CD5+/CD23+ B cells in the blood, bone marrow, lymph nodes and spleen [1, 2, 3]. Lots of mechanisms involved in leukemic transformation are reported. The deletion of specific micro-RNA genes leads to the resistance of B lymphocytes towards apoptosis [1, 4]. The B cell receptor (BCR) signaling plays an important pathogenic role because of BCR-dependent survival of leukemic lymphocytes [5]. Additionally, the accessory cells of microenvironment can promote leukemic cell growth. BCR and chemokine receptors were reported to allow lymphocytes to be localized in lymphoid tissues and to form the CLL microenvironment [6].

Clinical course and prognosis of CLL are reported as extremely variable. Some persons with benign course never require therapy and die because of other than leukemic ones causes. In others the disease is aggressive and treatment is started soon after diagnosis. Many prognostic factors are used to predict clinical outcome of CLL patients. The clinically important ones are connected with the biology of the disease -the mutation status of the variable segment of immunoglobulin heavy chain genes (IgVH)), ZAP-70 and CD38 expression as indicators for IgVH mutations, as well as gene and genomic abnormalities [7, 8, 9, 10].

Red blood cell distribution width (RDW) is a quantitative expression of the size heterogeneity of peripheral blood erythrocytes and is being usually automatically reported by laboratory blood analyzers in a complete blood cell count panels. RDW is presented as coefficient of variation (cv) of the mean value of erythrocytes volume (Coulter volume). Recently, the role of RDW as a prognostic marker for infections, such as severe sepsis, pneumonia, infections caused by gram-negative bacteria and necrotizing fasciitis was described [11, 12, 13, 14]. An elevated RDW unrelated of hemoglobin level, is connected with severe morbidity and mortality in the case of cardiovascular diseases, in kidney and pulmonary disorders [15, 16, 17, 18, 19]. RDW as a prognostic factor in hematological diseases was reported in patients with multiple myeloma [20]. Furthermore, RDW seems to be strong predictor of longevity, including all causes of death, with no apparent relationship to a particular disease, for people of age 45 and older [21].

The presented article aimed to analyze the possible role of RDW as a marker of prognosis in patients with CLL. Such a role of this parameter was expected because of the impairment of immunity in CLL patients and the fact that RDW is believed to reflect the general state of the patients and comorbidities related to their age, cardiovascular abnormalities or other infections.

## RESULTS

### Patients characteristics

**Figure 1:**
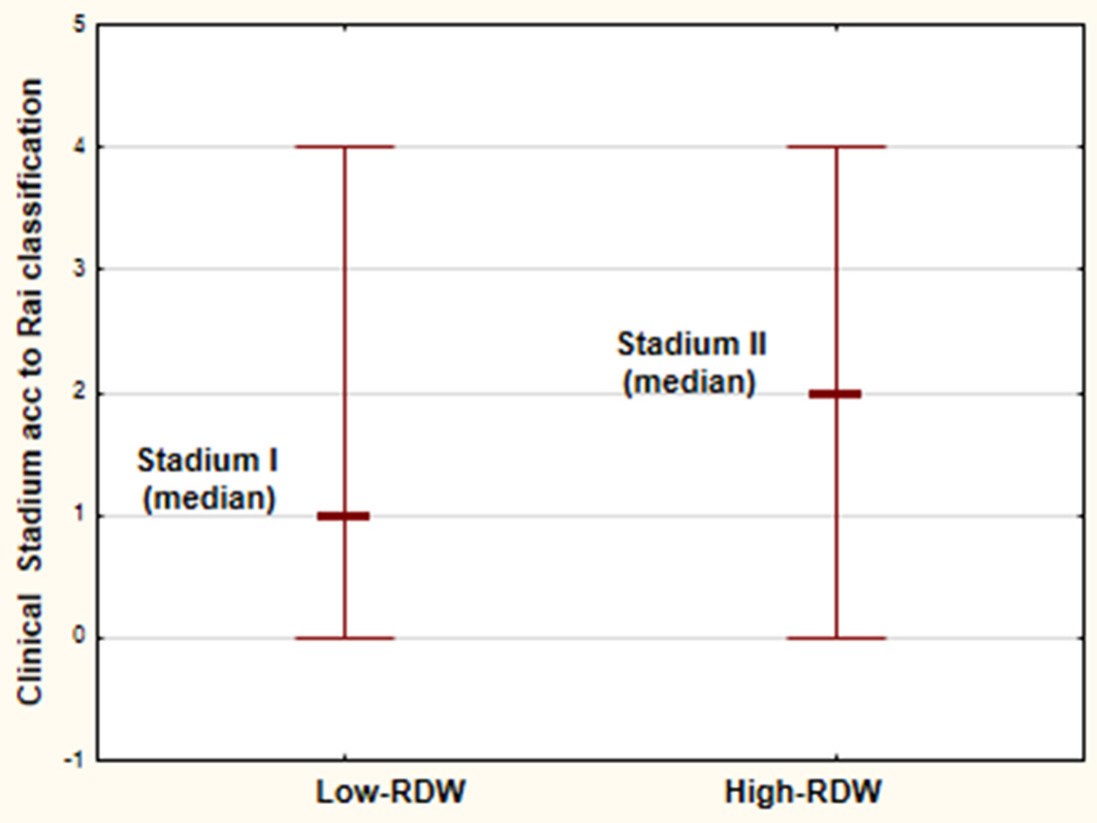
Clinical stadium according to Rai classification [30] of the analyzed CLL patients in High-RDW group (RDW > 14.5%) and Low-RDW group (RDW < 14.5%)

A total number of 66 previously untreated persons with CLL diagnosis were analyzed. The median age was 63 years, and 60% were males. Clinical characteristics of entire group of patients enrolled into the study is shown in Table 1. The median of pre-treatment RDW level was 13.5%, and the values ranged from 8.7 to 22.6%, while the normal ranges are established between 11.5% and 14.5%. The 14.5% cut-off was used for analysis as first selection. Additionally, we performed the receiver operating characteristic (ROC) curve assessment of RDW for ZAP-70 positive/negative, CD38 positive/negative, clinical classification early/advanced and cytogenetic risk standard/high, respectively, tofind another significant cut-off points for RDW values. The estimated RDW cut-off point for ZAP-70 was 12.7%, for CD38–13.7%, for clinical stadium was 14% and for cytogenetic risk groups–13.5%. However, the survival analysis has shown no significant differences in groups stratified according to these cut-offs. Thus, the selection of a 14.5% cut off for RDW positivity was chosen for final analysis. The analyzed subjects were classified as high-RDW (> 14.5%) and low-RDW (< 14.5). Table 2 presents clinical and laboratory parameters of persons stratified according to the RDW. High-RDW group included older persons comparing to low-RDW group, however the differences were not statistically significant. There were no statistically significant differences between the analyzed groups regarding white blood cell count and lymphocytosis, platelet count, lactate dehydrogenase level, β2-microglobulin level, presence of constitutive symptoms, lymphadenopathy or splenomegaly. Patients of high-RDW group were at more advanced stages when compared to low-RDW group (Figure 1).

**Table 1:** Clinical characteristics of analyzed CLL patients

Characteristics	Median (range)	Number of patients/percentage
**Sex**		
Female		30
Male		36
**Age**	63 (38–85)	
**Rai stadium**		
0		31%
I		17%
II		30%
III		8%
IV		14%
**CD38 expression**		
**Negative**		56%
**Positive**		44%
**ZAP-70 expression**		
Negative		68%
Positive		32%
**Cytogenetics**		
Low-risk		60%
High-risk		40%
**First-line therapy**		
Fludarabine-based regimens		30
Chlorambucil+/−Prednisone		7
Bendamustine		1

**Table 2:** Clinical parameters of analyzed CLL patients of Low-RDW (< 14.5%) and High-RDW (> 14.5%) groups

	Low - RDW Group	High -RDW Group	*p*
**Age (median)**	59	63	NS
**Lymphocytosis (10^9^/L)**	48 ± 43.5	75.7 + 60.3	NS
**Hemoglobin (g%)**	12.8 ± 1,7	11.4 ± 1.8	*p* < 0.05
**Platelets (10^9^/L)**	157 ± 75,2	140 ± 70.6	NS
**LDH (IU/L)**	370 ± 126	408 ± 145	NS
β**2-microglobulin (mg/dl)**	2.34 ± 0.7	5.44 ± 2.7	NS

Statistical significance is indicated (p values); NS – not significant.

**Figure 2:**
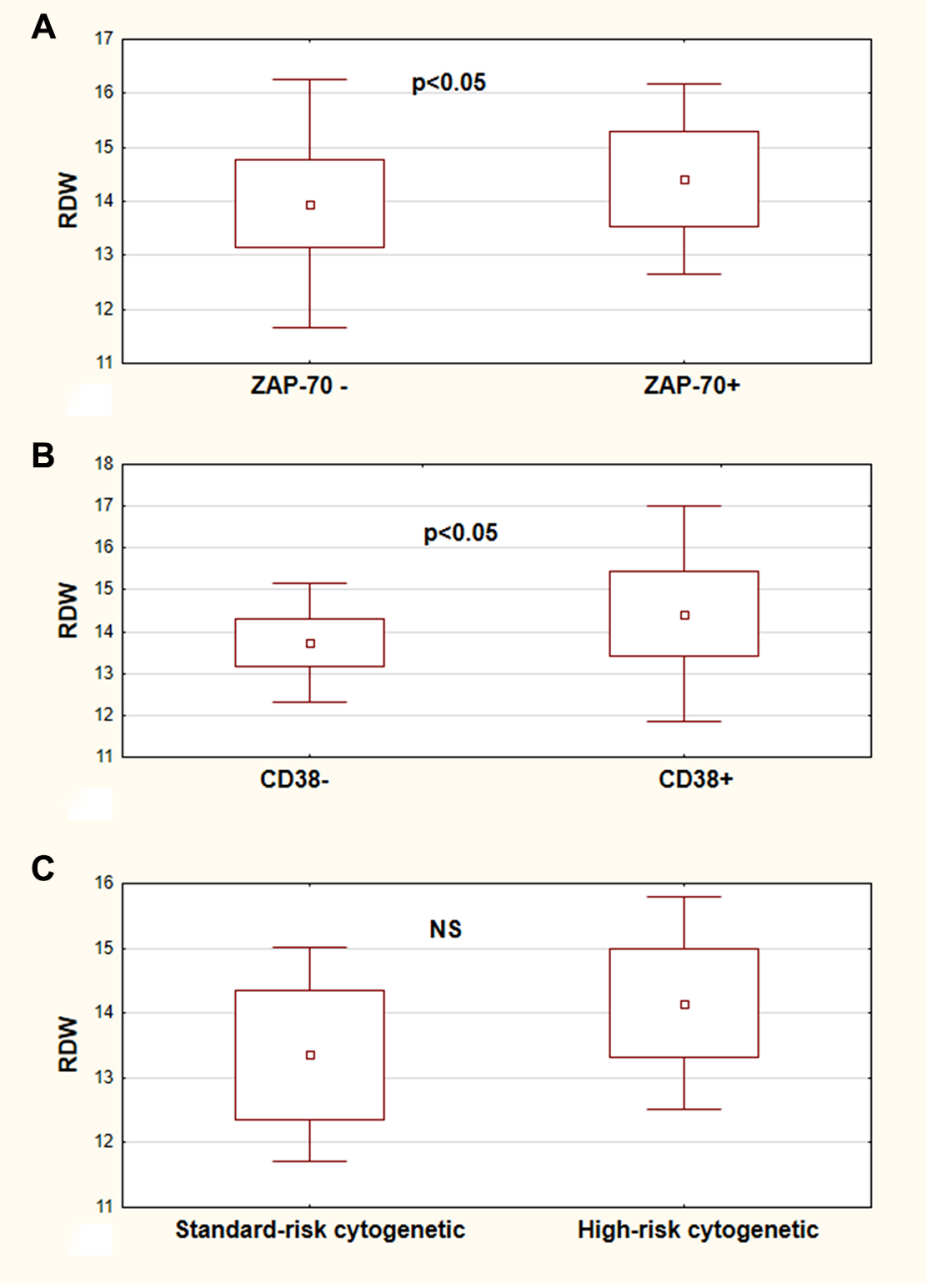
RDW values in CLL risk groups RDW values in ZAP-70 negative (ZAP-70-) and ZAP-70 positive (ZAP–70+) CLL patients (**A**). RDW values of CLL patients in CD38 negative (CD38−) and CD38 positive (CD38+) group (**B**). RDW values of analyzed patients in the standard-risk cytogenetic group (del13q14.3, trisomy 12, or no changes detected) and in the high-risk cytogenetic group (17p13.1 or 11q22.3) (**C**). All graphs show mean ± standard deviation. NS not statistically significant.

The patients were divided into two groups according to ZAP-70 and CD38 expression into positive group with ZAP-70 > 20% and CD38 > 20%, respectively and the negative group with ZAP-70 < 20% and CD38 < 20%, respectively. According to cytogenetic abnormalities the group of high-cytogenetic risk (del17p and del11q) and standard-cytogenetic risk group (trisomy of chromosome 12, del13q, no cytogenetic abnormalities) were distinguished. The RDW values were compared in the above groups. RDW was statistically significant higher in ZAP-70 positive and CD38 positive patients in comparison to ZAP-70 negative and CD38 negative subjects, respectively. In reference to cytogenetic changes, the difference were not statistically significant, however RDW was higher in high-cytogenetic risk group than in standard-cytogenetic risk one (Figure 2).

### RDW assessment in regard to clinical outcomes

**Figure 3:**
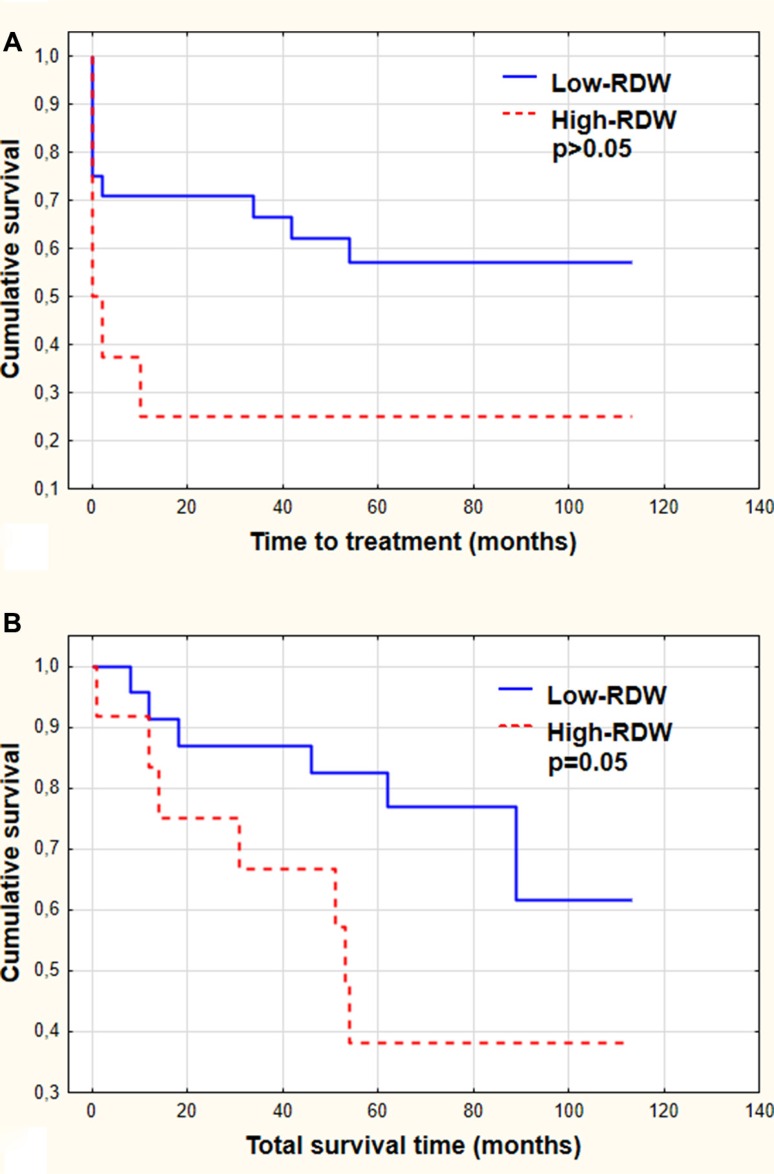
Time to treatment (A) and total survival time (B) demonstrated by Kaplan-Meier curves The patients were stratified according to RDW level (Low-RDW group versus High-RDW group). Statistical significance is indicated.

The clinical outcome data were analyzed in the group of patients who had to start the treatment (38 out of 66 patients). The difference in time to treatment between low -RDW group and high-RDW group was observed, however it was not statistically significant. The median time to treatment in low-RDW group was not reached (58% probability to survive 5 years without therapy) and it was 2 months in high-RDW group (*p* > 0.05). There were no statistically significant differences in response to chemotherapy in the group of analyzed patients. The median overall survival time in low-RDW group was not reached (the probability to survive 5 years was 77%), while in high-RDW patients it was 52 months. This difference was at the border of statistical significance (*p* = 0.05). The Kaplan–Meier estimates of time to treatment and overall survival time are illustrated in Figure 3. In multivariate Cox proportional hazard regression analysis of RDW plus other prognostic factors: CD38 expression, ZAP-70 expression and group of cytogenetic risk (not simple cytogenetic abnormalities), the RDW level and ZAP-70 expression were found to be the independent predictors of shorter survival (*p* = 0.04 and *p* = 0.03, respectively).

In the group of treated patients we analyzed RDW values longitudinally in particular subjects in different points such as the moment of diagnosis, the disease progression and the end of chemotherapy. There was no significant differences in RDW as the disease progress. In most of cases RDW remained in the same group of stratification (low-RDW or high-RDW). Thus, RDW seems to be stable, not time-dependent prognostic marker.

## DISCUSSION

An elevated RDW has been found as a poor prognostic factor in several diseases. Specifically, it is associated with high rate of morbidity and mortality in the case of patients with chronic heart failure [22, 23, 24]. Likewise, it is a marker of poor prognosis for patients with infectious diseases [25, 26]. However, there are relatively few reports focusing on RDW in the area of oncology. It is known that increase in RDW level indicates the presence of severe systemic inflammatory state that can worsen course of neoplastic diseases. RDW level can be influenced by anemia, which may worsen the course of neoplastic diseases as well. RDW appears to reflect the general condition of the patient, including such comorbidities as age, cardiovascular diseases and inflammation. It was reported that RDW was higher in women with aggressive breast cancer and is correlated with shorter survival in persons with lung cancer [27, 28, 29].

In the field of hematology the article by Lee et al. [20] focused on assessment of RDW in patients with symptomatic multiple myeloma. The observation that RDW level higher than normal at the moment of diagnosis was in correlation with more advanced disorder and poor prognosis prompted the authors to suggest that it could provide a novel and directly available predictor of severity of the disease. In fact, RDW may reflect both the inflammatory status as well as the patient's global condition [20].

The above observations induced us to analyze the prognostic role of RDW in CLL. The obtained results have shown that indeed RDW may be considered a prospective prognostic biomarker in this disease. The present data demonstrate that RDW is in correlation with other prognostic factors such as clinical stadium of the disease, expression of ZAP-70 and CD38. Anemia can be a symptom of CLL showing the most advanced stage of disease. In the presented study we have pointed that hemoglobin level is in correlation with RDW, but other CLL parameters such as ZAP-70 and CD38 expression have also changed according to RDW stratification It should be noted that anemia in neoplastic diseases like CLL does not simply reflect a decrease in peripheral blood erythrocytes number but may be a parameter of inflammation status as well, being associated with impaired iron release from reticuloendothelial macrophages [20].

Clinical stadium of the disease for many years belongs to the most important prognostic factors in CLL. Nowadays, two staging methods - the Rai [30] and the Binet system [31] co-exist and are widely used in clinical practice. Early stage 0 disease according to Rai classification describes patients with lymphocytosis detected in the peripheral blood and bone marrow. Patients with lymphocytosis and enlarged lymph nodes are classified at stadium I and those with splenomegaly at stadium II. High risk disease includes patients with leukemia-related anemia or thrombocytopenia (stage III and IV, respectively) [30, 32]. The Binet staging system is based on the number of involved areas of lymph nodes or organomegaly and on whether there is anemia or thrombocytopenia [31, 32]. In general practice, newly diagnosed patients with asymptomatic early-stage disease should be observed without treatment. The patients at intermediate and high risk usually have to start treatment, however some of them can be observed without therapy until disease progression [22, 32]. Among the biological CLL prognostic markers the IgVH mutation status or its surrogate markers (ZAP-70 and CD38 expression) as well as genomic aberrations may identify the subjects with more aggressive leukemia and poor prognosis [7, 33]. The correlations between RDW level and some prognostic markers of CLL detected in our analysis may indicate the role of RDW assessment in prognosis of CLL patients.

Further analysis performed in our study was focused on association between RDW level and clinical outcomes. We detected no differences between the time of the initiating of the therapy as far as RDW level was concerned, however the differences in overall survival time were significant. As mentioned, strong association between RDW and longevity has been shown for subjects 45 years and older [21]. This association remains strong after discounting all types of red blood cell diseases [21]. To explain this occurrence it was proposed that persistent signaling along the IGFs/mTOR pathway is one of the key factors that play a role in affecting the RDW and mortality [34–36]. Specifically, there are the data that IGF-1/mTOR signaling enhances erythropoiesis by activation of erythropoietin [36, 37] and affects the final steps of erythroid lineage maturation [38]. The IGFs/mTOR also strikingly influences the cell size as shown for 32D- originated myeloid cells that are 50% smaller while having deleted IGF-1 receptor [39]. It may be possible therefore that persistent activation of erythropoiesis through IGF-1/mTOR results in heterogeneity of red cell sizes due to their higher turnover rates. On the other hand the elevated level of GH/IF-1/mTOR signaling is considered to be the primary cause of aging and age-associated mortality [39, 40]. Given the above it may be expected that RDW may be a useful marker of the constitutive level of IGF-1 signaling, the critical factor accountable for metabolic aging and longevity [21]. It is possible, therefore, that the presently observed correlation between mortality and RDW of the CLL patients is affected by their metabolically driven (growth factor - IGF-1/mTOR)- rather than chronological- aging.

Attempts have been recently made to apply mTOR inhibitors in treatment of leukemia, including CLL [41, 42, 43]. Along the rationale presented above the patients with high level of RDW are expected to have an increased persistent level of IGF-1/mTOR signaling. Within the framework of personalized therapy, these CLL patients therefore would be expected to be more sensitive to the treatment with mTOR inhibitors.

In conclusion, the presented study underscores the potential role of RDW as a prognostic factor in CLL. Elevated RDW level at diagnosis was associated with advanced disease and the presence of other poor prognostic factors. It was also connected with life expectancy indicating shorter survival time of patients with elevated RDW. These results thus indicate that RDW may be considered as a simple and easily available prognostic factor. Such a role of RDW could be anticipated considering that RDW may reflect the overall conditions of the patients and be associated with their longevity (21). Further studies, however are required to ascertain specifically molecular mechanism(s) linking the increased RDW with poor prognosis of CLL.

## MATERIALS AND METHODS

### Patients

We performed a retrospective analysis of the medical records of a group of patients diagnosed with CLL in our Department. Diagnosis of the disease was made on the basis of clinical examination, morphological and immunological criteria. The persons who had complete blood count tests results available with the reported RDW before treatment were enrolled into the study; those who had received a blood transfusion within the previous six months were excluded. Finally sixty six patients were analyzed. We used a coding system to assure the anonymity of the patients enrolled into the study.

### Measurement of RDW

RDW was measured with use of automatic analyzer Sysmex XS-1000i and was reported as a coefficient of variation of the mean red blood cell volume. The reference range for RDW was 11.5% to 14.5%.

### Data collection

Firstly, the age, sex and data of CLL diagnosis were recorded. The laboratory findings beside RDW collected at the moment of diagnosis including white blood cell count and lymphocyte count, hemoglobin level, platelet count, lactate dehydrogenase level, β2microglobulin level, presence of constitutive symptoms, lymphadenopathy or splenomegaly were then reviewed. All patients were also characterized according to expression of ZAP-70 and CD 38 that were assessed with flow cytometry method and according to presence of cytogenetic abnormalities assessing with FISH (fluorescence *in situ* hybridization) method. The next step of analysis was focused on therapy data. We analyzed time from diagnosis to starting of the therapy, chemotherapy regimens ordered and chemotherapy outcome. We used the criteria of response to treatment proposed by WG- IWCLL in 2008 [44] based on WG- NCI criteria from1996 [45]. According to these criteria, complete response requires the absence of symptoms and organomegaly, normal complete cell counts of peripheral blood and less than 30% of lymphocytes in bone marrow for at least 2 months. When size of the lymph nodes, spleen and liver, together with the peripheral blood data, were at least 50% better than pre-treatment values, the partial response was achieved. Other patients were considered non-responders. As the last step of analysis time of overall survival was analyzed.

### Statistical analysis

The collected data were analyzed using STASTICA 12 Software by StatSoft Inc. The Mann-Whitney and Wilcoxon tests were used for groups comparison. The Kaplan–Meier method was employed to calculate the survival analysis. Multivariate analysis of independent clinical factors for survival was tested by the Cox proportional hazard regression model. The assessment of cut-off points for RDW were assessed by drawing of receiver operating characteristic (ROC) curves using Medical Bundle for STATISTICA 12. Differences were considered statistically significant when *p* values were < 0.05.

## References

[1] Calligaris-Cappio F, Hamblin TJ (1999). B-Cell Chronic Lymphocytic leukemia: A Bird of a Different Feather. J Clin Oncol.

[2] Chiorazzi N, Rai KR, Ferrarini M (2005). Chronic lymphocytic leukemia. N Engl J Med.

[3] Hamblin TJ, Oscier DG (1997). Chronic lymphocytic leukemia: the nature of the leukemic cells. Blood Rev.

[4] Calin GA, Dumitru CD, Shimizu M, Bichi R, Zupo S, Noch E, Aldler H, Rattan S, Keating M, Rai K, Rassenti L, Kipps T, Negrini M (2002). Frequent deletions and downregulation of micro-RNA genes miR15 and miR16 at 13q14 in chronic lymphocytic leukemia. Proc Natl Acad Sci USA.

[5] Burger1 JA, Nicholas Chiorazzi N (2013). B cell receptor signaling in chronic lymphocytic leukemia. Trends Immunol.

[6] Byrd JC, Jones JJ, Woyach JA, Johnson AJ, Flynn JM (2014). Entering the Era of Targeted for Chronic Lymphocytic Leukemia: Impact on the Practicing Clinician. J Clin Oncol.

[7] Stilgenbauer S (2006). Chromic lymphocytic leukemia: genetics for predicting outcome. Hematology.

[8] Crespo M, Bosch F, Villamor N, Bellosillo B, Colomer D, Rozman M, Marcé S, López-Guillermo A, Campo E, Montserrat E (2003). ZAP-70 expression as a surrogate for immunoglobulin-variable-region mutations in chronic lymphocytic leukemia. N Engl J Med.

[9] Hamblin TJ, Davis Z, Gardiner A, Oscier DG, Stevenson FK (1999). Unmutated Ig V(H) genes are associated with a more aggressive form of chronic lymphocytic leukemia. Blood.

[10] Oscier DG, Thompsett A, Zhu D, Stevenson FK (1997). Differential rates of somatic hypermutation in V(H) genes among subsets of chronic lymphocytic leukemia defined by chromosomal abnormalities. Blood.

[11] Weng CL, Wang CH, Chen IC, Hsiao KY, Chang KP, Wu SY, Shih HM (2014). Red cell distribution width is an independent predictor of mortality in necrotizing fasciitis. Am J Emerg Med.

[12] Braun E, Domany E, Kenig Y, Mazor Y, Makhoul BF, Azzam ZS (2011). Elevated red cell distribution width predicts poor outcome in young patients with community acquired pneumonia. Crit Care.

[13] Ku NS, Kim HW, Oh HJ, Kim YC, Kim MH, Song JE, Oh DH, Ahn JY, Kim SB, Jeong SJ, Han SH, Kim CO, Song YG (2012). Red blood cell distribution width is an independent predictor of mortality in patients with gram-negative bacteremia. Shock.

[14] Jo YH, Kim K, Lee JH, Kang C, Kim T, Park HM, Kang KW, Kim J, Rhee JE (2013). Red cell distribution is a prognostic factor in severe sepsis and septic shock. Am J Emerg Med.

[15] Tseliou E, Terrovitis JV, Kaldara EE, Ntalianis AS, Repasos E, Katsaros L, Margari ZJ, Matsouka C, Toumanidis S, Nanas SN, Nanas JN (2014). Red blood cell distribution width is a significant prognostic marker in advanced heart failure, independent of hemoglobin levels. Hellenic J Cardiol.

[16] Tonelli M, Sacks F, Arnold M, Moye L, Davis B, Pfeffr M (2008). Relation between red blood cell distribution width and cardiovascular event rate in people with coronary disease. Circulation.

[17] Arbel Y, Weitzman D, Raz R, Steinvil A, Zeltser D, Berliner S, Chodick G, Shalev V (2014). Red blood cell distribution width and the risk of cardiovascular morbidity and allcause mortality. A population-based study. Thromb Haemost.

[18] Oh HJ, Park JT, Kim JK, Yoo DE, Kim SJ, Han SH, Kang SW, Choi KH, Yoo TH (2012). Red blood cell distribution width is an independent predictor of mortality in acute kidney injury patients treated with continuous renal replacement therapy. Nephrol Dial Transplant.

[19] Solak Y, Yilmaz MI, Saglam M, Caglar K, Verim S, Unal HU, Gok M, Demirkaya E, Gaipov A, Kayrak M, Cetinkaya H, Eyileten T, Turk S (2014). Red cell distribution width is independently related to endothelial dysfunction in patients with chronic kidney disease. Am J Med Sci.

[20] Lee H, Kong SY, Sohn JY, Shim H, Youn HS, Lee S, Kim HJ, Eom HS (2014). Elevated red blood cell distribution width as a simple prognostic factor in patients with symptomatic multiple myeloma. Biomed Res Int.

[21] Patel KV, Ferruci L, Ershler WB, Longo DL, Guralnik JM (2009). Red cell distribution width and the risk of death in middle-aged and older adults. Arch Intern Med.

[22] Felker GM, Allen LA, Pocock SJ, Shaw LK, McMurray JJ, Pfeffer MA, Swedberg K, Wang D, Yusuf S, Michelson EL, Granger CB, CHARM Investigators (2007). Red cell distribution width as a novel prognostic marker in heart failure: data from the CHARM Program and the Duke Databank. J Am Coll Cardiol.

[23] Allen LA, Felker GM, Mehra MR, Chiong JR, Dunlap SH, Ghali JK, Lenihan DJ, Oren RM, Wagoner LE, Schwartz TA, Adams KF (2010). Validation and potential mechanisms of red cell distribution width as a prognostic marker in heart failure. J Card Fail.

[24] Akin F, Köse N, Ayça B, Katkat F, Duran M, Uysal OK, Arinc H (2013). Relation between red cell distribution width and severity of coronary artery disease in patients with acute myocardial infarction. Angiology.

[25] Braun E, Kheir J, Mashiach T, Naffaa M, Azzam ZS (2014). Is elevated red cell distribution width a prognostic predictor in adult patients with community acquired pneumonia?. BMC Infect Dis.

[26] Braun E, Domany E, Kenig Y, Mazor Y, Makhoul BF, Azzam ZS (2011). Elevated red cell distribution width predicts poor outcome in young patients with community acquired pneumonia. Crit Care.

[27] Seretis C, Seretis F, Lagoudianakis E, Gemenetzis G, Salemis NS (2013). Is red cell distribution width a novel biomarker of breast cancer activity? Data from a pilot study. J Clin Med Res,.

[28] Warwick R, N Mediratta N, Shackcloth M, Shaw M, McShane J, Poullis M (2014). Preoperative red cell distribution width in patients undergoing pulmonary resections for non-small-cell lung cancer. Eur J Cardiothorac Surgvol.

[29] Koma Y, Onishi A, Matsuoka H, Oda N, Yokota N, Matsumoto Y, Koyama M, Okada N, Nakashima N, Masuya D, Yoshimatsu H, Suzuki Y (2013). Increased red blood cell distribution width associates with cancer stage and prognosis in patients with lung cancer. PLoS ONE.

[30] Rai KR, Sawitsky A, Cronkite EP, Chanana AD, Levy RN (1975). Pasternack BS Clinical staging of chronic lymphocyticleukemia. Blood.

[31] Binet JL, Auquier A, Dighiero G, Chastang C, Piguet H, Goasguen J, Vaugier G, Potron G, Colona P, Oberling F, Thomas M, Tchernia G, Jacquillat C (1981). A new prognostic classification of chronic lymphocytic leukemia derived from a multivariate survival analysis. Cancer.

[32] Hallek M (2013). Chronic lymphocytic leukemia: 2013 update on diagnosis, risk stratification and treatment. Am J Hematol.

[33] Gribben JG (2008). Molecular profiling in CLL. Hematology Am Soc Hematol Educ Program.

[34] Halicka HD, Zhao H, Li J, Lee Y-S, Hsieh T-C, Wu JM, Darzynkiewicz Z (2012). Potential anti-aging agents suppress the level of constitutive DNA damage- and mTOR- signaling. Aging (Albany).

[35] Darzynkiewicz Z, Zhao H, Halicka HD, Li J, Lee Y-S, Hsieh T-C, Wu J (2014). In search of anti-aging modalities: evaluation of mTOR- and ROS/DNA damage- signaling by cytometry. Cytometry A.

[36] Kim I, Kim CH, Yim YS, Ahn YS (2008). Autocrine function of erythropoietin in IGF-1-induced erythropoietin biosynthesis. Neuroreport.

[37] Kling PJ, Taing KM, Dvorak B, Woodward SS, Philipps AF (2006). Insulin-like growth factor-I stimulates erythropoiesis when administered enterally. Growth Factors.

[38] Ratajczak J, Zhang Q, Pertusini E, Wojczyk BS, Wasik MA, Ratajczak MZ (1998). The role of insulin (INS) and insulin-like growth factor-1 (IGF-I) in regulating human erythropoiesis. Studies *in vitro* under serum-free conditions—comparison to other cytokines and growth factors. Leukemia.

[39] Anisimov VN, Bartke A (2013). The key role of growth hormone-insulin- GF-1 signaling in aging and cancer. Crit Rev Oncol Hematol.

[40] Blagosklonny MV (2013). Rapamycin extends life- and health span because it slows aging. Aging (Albany NY).

[41] Bertacchini J, Heidari N, Mediani L, Capitani S, Shahjahani M, Ahmadzadeh A, Saki N (2015). Targeting PI3K/AKT/mTOR network for treatment of leukemia. Cell Mol Life Sci.

[42] Blunt MD, Carter MJ, Larrayoz M, Smith LD, Aguilar-Hernandez M, Cox KL, Tipton T, Reynolds M, Murphy S, Lemm E, Dias S, Duncombe A, Strefford JC (2015). The PI3K/mTOR inhibitor PF-04691502 induces apoptosis and inhibits microenvironmental signaling in CLL and the Eμ-TCL1 mouse model. Blood.

[43] Zent CS, Bowen DA, Conte MJ, LaPlant BR, Call TG (2015). Treatment of relapsed/refractory chronic lymphocytic leukemia/small lymphocytic lymphoma with everolimus (RAD001) and alemtuzumab: a Phase I/II study. Leuk Lymphoma.

[44] Cheson BD, Catovsky D, Caligaris-Cappio F, Dighiero G, Döhner H, Hillmen P, Keating MJ, Montserrat E, Rai KR, Kipps TJ, International Workshop on Chronic Lymphocytic Leukemia (2008). Guidelines for the diagnosis and treatment of chronic lymphocytic leukemia: a report from the International Workshop on Chronic Lymphocytic Leukemia updating the National Cancer Institute-Working Group 1996 guidelines. Blood.

[45] Cheson BD, Bennett JM, Grever M, Kay N, Keating MJ, O'Brien S, Rai KR (1996). National Cancer Institute-sponsored Working Group guidelines for chronic lymphocytic leukemia: revised guidelines for diagnosis and treatment. Blood.

